# Latent growth curve modeling of physical activity trajectories in a positive-psychology and motivational interviewing intervention for people with type 2 diabetes

**DOI:** 10.1080/21642850.2022.2104724

**Published:** 2022-08-04

**Authors:** Rachel A. Millstein, Julia Golden, Brian C. Healy, Hermioni L. Amonoo, Lauren E. Harnedy, Alba Carrillo, Christopher M. Celano, Jeff C. Huffman

**Affiliations:** a Department of Psychiatry, Massachusetts General Hospital, Boston, MA, USA; b Harvard Medical School, Boston, MA, USA; c School of Medicine, University of Connecticut, Farmington, CT, USA; d Department of Neurology, Brigham and Women’s Hospital, Boston, MA, USA; e Department of Psychiatry, Brigham and Women’s Hospital, Boston, MA, USA; f Department of Psychosocial Oncology, Dana-Farber Cancer Institute, Boston, MA, USA

**Keywords:** Type 2 diabetes, physical activity, latent growth model, longitudinal model, positive psychology

## Abstract

Background: Physical activity is critical for preventing and treating Type 2 diabetes (T2D). It is important to identify different profiles of physical activity change among those participating in behavioral interventions to optimize intervention-person fit.

Methods: This study analyzes longitudinal trajectories of change in moderate-to-vigorous physical activity (MVPA) in a positive psychology (PP) and motivational interviewing (MI) intervention for T2D, using latent growth curve modeling (LGCM). Objective measures of MVPA were collected using accelerometers at three time points: pre-intervention, immediately post-intervention, and eight weeks post-intervention. LGCM analyses identified subpopulations of participants who responded similarly to the intervention and examined if sociodemographic, medical and psychosocial characteristics were associated with MVPA trajectories.

Results: Analyses included 47 participants with complete follow-ups: 49% male, 81% non-Hispanic white, average age 66.1 (*SD* = 10.1). Overall, 36% of the participants increased MVPA while 57% did not. LGCM identified three profiles with distinct MVPA trajectories. Profile 1 (‘Started Low, No Change’; 65.8% of participants) with a starting mean of 4.54 min of MVPA/day and decreased by −3.36 min. Profile 2 (‘Moderate-High Start, Minimal Change,’ 27.4% of participants) and had a starting mean of 22.86 min/day of MVPA with an average increase of 1.03 min. Profile 3 (‘Moderate Start, Ended High’; 6.8% of participants), had a starting mean of 7.33 min MVPA/day, and increased by 28.4 min. Being male, younger, having fewer medical and psychiatric comorbidities were associated with increases in MVPA.

Conclusions: This secondary analysis detected three distinct physical activity profiles during and after a PP-MI intervention. Future interventions can target individuals with characteristics that showed the greatest benefit and add additional supports to people in groups that did not increase physical activity as much. These findings show a need for targeted and sustained behavior change strategies during and after physical activity interventions.

**Trial registration:** ClinicalTrials.gov; identifier: NCT03001999.

**Abbreviations**
BEHOLD: boosting emotional well-being and happiness in outpatients living with diabetes
BEHOLD-8: boosting emotional well-being and happiness in outpatients living with diabetes 8-week
BEHOLD-16: boosting emotional well-being and happiness in outpatients living with diabetes 16-week
T2D: type 2 diabetes
PP: positive psychology
MI: motivational interviewing
MVPA: moderate-to-vigorous physical activity
LGCM: latent growth curve model
IPAQ: International Physical Activity Questionnaire
ADA: American Diabetes Association
GLLAMM: generalized linear latent and mixed models
AIC: Akaike information criterion
BIC: Bayesian information criterion
ES: effect size

## Introduction

Type 2 diabetes (T2D) affects 10.5% of US adults (US Centers for Disease Control and Prevention, 2021a) and 6.3% of adults worldwide (Khan et al., 2020), and the prevalence is projected to increase (Khan et al., 2020). Physical activity (PA) is a critical lifestyle behavior to reduce mortality risk in patients with T2D and improve physical and emotional functioning (Avery, Flynn, van Wersch, Sniehotta, & Trenell, 2012). However, similar to the US population, more than 75% of adults with T2D do not meet recommended physical activity guidelines of at least 150 min/week of moderate physical activity (Thomas, Alder, & Leese, 2004; US Centers for Disease Control and Prevention, 2021a; US Centers for Disease Control and Prevention, 2021b).

Physical activity interventions for people with T2D can be effective for glycemic control and overall health, though they tend to be intensive, have limited description of theoretical bases, and can be difficult to implement in clinical settings (Avery et al., 2012). Physical activity interventions can range from in-person to virtual, individual to group-based, and at home to community settings (van der Bij, Laurant, & Wensing, 2002). While effects may range, overall, changes are often small and may not be sustained over time (van der Bij et al., 2002).

Despite ongoing efforts to promote physical activity in patients with chronic diseases such as T2D, one-size-fits-all behavioral interventions may not be universally effective (van Sluijs et al., 2005). There has been a more recent initiative to tailor or target physical activity interventions to increase uptake and engagement in specific communities (Short, James, Plotnikoff, & Girgis, 2011). Findings have shown that personally relevant interventions, such as those tailored for women and specific racial or ethnic groups have been more successful than those targeting a broad audience (Bock, Jarczok, & Litaker, 2014; Dunton & Robertson, 2008 Yap & Davis, 2008;). However, there has been less of an emphasis on tailoring interventions for subgroups of people with T2D (Clark, Hampson, Avery, & Simpson, 2004). Thus there is a need to understand how different populations engage with behavioral intervention, which can lead to more stratification or tailoring to the needs of distinct subpopulations.

Because it is important to identify groups of people for whom interventions may be more or less successful, latent growth curve modeling (LGCM) (Duncan & Duncan, 2009; Roesch et al., 2009) is a useful tool in intervention development. LGCM can be used to identify groups or subpopulations with similar treatment responses within a larger study population. Unlike conventional growth models, which assume that one growth trajectory can represent an entire population, LGCM accounts for between-person differences and can flexibly classify participants into different growth trajectories based on unobserved characteristics (Duncan & Duncan, 2009). Accordingly, the purpose of this secondary analysis was to identify subgroups of participants in two behavioral physical activity interventions for T2D and identify groups for whom the interventions were more and less helpful. The Boosting Emotional well-being and Happiness in Outpatients Living with Diabetes 8-week and 16-week (BEHOLD-8 and BEHOLD-16) were randomized controlled trials to promote physical activity in patients with T2D (Huffman et al., 2020; Huffman et al., 2021; Zambrano et al., 2020). Both BEHOLD-8 and BEHOLD-16 were previously found to be feasible, acceptable, and led to overall improvements in physical activity, with small to medium effect size (Huffman et al., 2020; Huffman et al., 2021). With these promising findings at the intervention group level, it becomes important to understand how subgroups responded to the interventions.

The goals of this secondary analysis were to explore the presence and trajectories of latent growth curves in objectively measured moderate-vigorous physical activity (MVPA) among BEHOLD-8 and -16 participants. These analyses were of particular use in the current study as they can help to determine for whom (i.e. which subgroups) the intervention was most beneficial, if participants changed their PA over the course of the study, and if so, the characteristics of the groups.

## Methods

The BEHOLD-8 and BEHOLD-16 randomized controlled trials examined the impact of a combined positive psychology (PP) and motivational interviewing (MI) phone-delivered intervention on improving PA in patients with T2D. The combination of PP with MI (PP-MI) was tested compared to time- and attention-matched control conditions, respectively (Huffman et al., 2020; Huffman et al., 2021; Zambrano et al., 2020). Both strategies employ straightforward activities that can enhance motivation for health behavior change (e.g. physical activity, smoking cessation) (Gorman et al., 2014; Watson, Clark, & Tellegen, 1988). PP promotes positive cognitive and emotional states such as vitality and optimism (Watson et al., 1988) that can help people increase engagement in physical activity and other health behaviors (Bjelland, Dahl, Haug, & Neckelmann, 2002; Charlson, Pompei, Ales, & MacKenzie, 1987). MI is an evidence-based, patient-centered strategy that seeks to enhance a patient’s intrinsic desire to change (Gorman et al., 2014).

### Participants

Participants, enrolled between June 2017 and May 2019 (follow-ups complete in October 2019), were primary care patients from an urban academic medical center who: (1) met American Diabetes Association (ADA) criteria for T2D (American Diabetes Association, 2013) (e.g. hemoglobin A1c [A1C] ≥6.5%, fasting glucose ≥126 mg/dl) and (2) met criteria for low baseline physical activity, defined as ≤150 min of moderate-to-vigorous physical activity (MVPA) per week (i.e. not meeting national recommendations as measured by the International Physical Activity Questionnaire (IPAQ)) (Lee, Macfarlane, Lam, & Stewart, 2011). Patients were *excluded* if they had: (1) cognitive impairment precluding consent or meaningful participation, (2) lack of phone availability, (3) inability to read/write in English, (4) additional medical conditions (e.g. severe arthritis) that would make physical activity very difficult, or (5) current enrollment/participation in lifestyle intervention programs (e.g. cardiac rehabilitation), clinical trials or other research studies. Inclusion and exclusion criteria were identical for both BEHOLD-8 and 16 trials.

### Screening and randomization

Medical study staff identified and screened potential participants for eligibility using the hospital’s electronic health record data registry. Once approved by medical providers, patients were randomly assigned to BEHOLD-8 or BEHOLD-16 and randomly assigned to conditions within each study (see Zambrano et al., 2020 for additional details).

In both studies, eligible participants completed an initial, in-person study visit (Time 1), at which they provided written informed consent, completed self-report outcome measures, and received an Actigraph GT3X+ waist-worn accelerometer (Actigraph Corp., Pensacola, FL) to wear for seven days before returning back to the hospital for randomization. Participants then attended a second in-person study visit during which they were randomly assigned a condition (PP-MI or control). Following condition assignment, participants met with a study interventionist (trained psychologist), who provided a condition-specific treatment manual, reviewed the first week’s material, and assigned an initial activity. All participants, regardless of condition, received a waist-worn, Omron HJ-520 pedometer to track physical activity. The remainder of all intervention content was delivered via phone.

#### Trials

**Figure 1.**
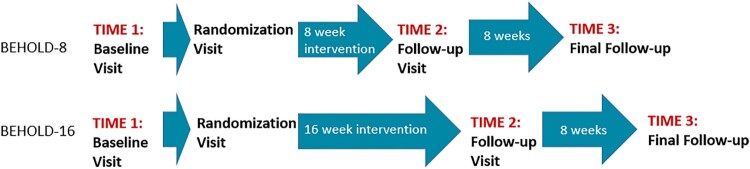
Study intervention and assessment timelines (BEHOLD-8 and 16).

*BEHOLD-8* (Huffman et al., 2020; Zambrano et al., 2020). The BEHOLD-8 trial was an eight-week intervention program consisting of eight sessions of PP-MI or MI-based health education (control) intervention. The control condition was attention-matched and focused on behavioral counseling for health education to assist participants in making changes to diet, physical activity, medication adherence, and overall diabetes self-care (see 15 for more details). Based on power calculations, 60 participants were randomized. Upon completion of the intervention, participants completed an in-person visit (Time 2) to complete the same self-report outcome measures as at baseline, and they received another accelerometer to wear for seven days. Finally, 16 weeks after the randomization visit, participants completed the same self-report outcome measures as at baseline (Time 3). They also wore a third accelerometer for seven days and mailed it back. See Figure 1 for intervention timelines.

*BEHOLD-16* (Huffman et al., 2021; Zambrano et al., 2020). The BEHOLD-16 trial was a 16-week intervention consisting of 14 sessions of PP-MI or MI-based time- and attention-matched health education control condition. The control condition had a parallel structure to the intervention and focused on four modules: diabetes self-care, medication adherence, diet, and physical activity (see 17 for more details). Based on power calculations, 70 participants were randomized. Enrollment and the first two in-person assessment visits were identical to BEHOLD-8. Upon completion of the intervention, participants completed another in-person visit (Time 2) to complete the self-report outcome measures from baseline, and they received another accelerometer to wear for seven days and mail back. Participants were allotted a three-week window upon reaching 16 weeks following the randomization visit in which to complete this follow-up visit. Finally, 24 weeks following the randomization visit, participants completed the same self-report outcome measures as at baseline (Time 3). They also wore a third accelerometer for seven days and mailed it back.

#### Interventions

The weekly PP-MI phone sessions lasted approximately 30–45 min and consisted of a PP component followed by a separate MI component that utilized MI principles (and goal-setting) to promote physical activity (Huffman et al., 2020; Huffman et al., 2021; Zambrano et al., 2020). The PP exercises were chosen based on the literature and the team’s prior work delivering PP interventions, and adapted specifically for patients with T2D (Celano et al., 2019; Huffman et al., 2017). Each PP session involved a weekly topic and practice (e.g. writing a letter of gratitude, using personal strengths), which the interventionist would review with the participant using the treatment manual. The MI portion of the session focused on a specific MI or PA topic (e.g. identifying pros and cons of increasing activity), and participants reviewed these topics with their interventionist using the treatment manual. Participants also set individualized physical activity goals for the upcoming week and reviewed the previous week’s goal with their interventionist. Both BEHOLD-8 and -16 sessions covered the same topics, but BEHOLD-16 spread the material over a longer period of time. See references (Huffman et al., 2020; Huffman et al., 2021; Zambrano et al., 2020) for more details regarding session content and structure.

#### Measures

*Objectively measured physical activity*: Participants wore Actigraph GT3X+ accelerometers (Actigraph, Pensacola, FL) to assess their objectively measured average MVPA per day and average steps per day. Participants were required to have a minimum of 8 h wear time of the Actigraph for 4+ days. To assess MVPA, we chose the commonly used cutoff for adult populations of 1952 counts/minute (Gorman et al., 2014; Zambrano et al., 2020). Participants wore the Actigraphs at three time points: (1) prior to the intervention (Time 1), (2) immediately following the intervention (Time 2), and (3) eight weeks following the intervention’s conclusion (Time 3; Figure 1). Average MVPA (minutes/day) and steps at each time point were found by dividing their total minutes of MVPA or total number of steps by the number of days the participant wore the Actigraph at that time point.

Findings from the parent studies indicated that PP-MI participants in the BEHOLD-8 trial had greater improvements in MVPA at eight weeks than the control condition by 13.05 min/day, and at 16 weeks, by 7.96 min/day (Huffman et al., 2020). For the BEHOLD-16 trial, PP-MI participants had small to medium effect size (ES) difference greater improvements in MVPA (ES difference = 0.34) and steps/day (ES difference = 0.76) at 16 weeks, with sustained but smaller intervention effects at 24 weeks (ES difference = 0.22–0.33) (Huffman et al., 2021).

*Demographics*: Participants provided demographic data (race, ethnicity, marital status, annual income, employment status, and educational status) through a baseline survey.

*Psychological measures*: Participants completed validated questionnaires to assess positive affect, optimism, and anxiety/depression at Time 1. Positive affect was measured using the 10-item positive subscale of the Positive and Negative Affect Schedule (PANAS) (Watson et al., 1988) (*α* in this sample = .90). Optimism was measured with the six-item Life Orientation Test-Revised (LOT-R) (Scheier, Carver, & Bridges, 1994) (*α* = .76). Depression and anxiety were measured with the 14-item Hospital Anxiety and Depression Scale (HADS-A and HADS-D) (Bjelland et al., 2002) subscales for depression and anxiety (*α* = .78 [depression] and *α* = .81 [anxiety]).

*Medical*: Medical records were reviewed by study team physicians at enrollment to collect data on comorbid conditions related to T2D, including neuropathy, nephropathy, hyperlipidemia, hypertension and coronary artery disease, and medical comorbidity was assessed using the age-adjusted Charlson Comorbidity Index (Charlson et al., 1987). Information about medications (non-insulin diabetes medications, insulin and antidepressant/anxiolytics) prescribed at enrollment was also recorded from medical chart reviews. Non-insulin diabetes medications included metformin and sulfonylureas. Additionally, participants completed validated questionnaires to assess pain and physical functioning at Time 1. Pain was measured using the Pain Disability Index (PDI) (Tait, Chibnall, & Krause, 1990), and physical functioning was measured using the Physical Function subsection of the Patient-Reported Outcomes Measurement Information System (PROMIS-physical function 20-item scale (Bartlett et al., 2015); *α* = .89).

#### Statistical analysis

These secondary analyses included participants in the PP-MI condition from either BEHOLD-8 or BEHOLD-16 who had physical activity data available at all three time points. Therefore, participants in the control condition and those missing data at any time point were removed from these analyses. There were no significant differences between those with complete physical activity data (included in analyses) and those with incomplete physical activity data (excluded from analyses) with the exception that there were more non-Hispanic White people in the included/completer group (Supplemental Table S1). Control group physical activity (MVPA) data were explored using Stata line graphs (xtline code) and using descriptive statistics. Due to the majority of participants showing no changes or patterns in activity, and the primary goal of determining intervention utility, only PP-MI data are included in the analyses shown here. The studies were combined in order to maximize sample size for this analysis, and because they contained similar intervention materials, despite the differing timeframes. All analyses were two-tailed and performed using Stata 14.2 and 16.0 (StataCorp: College Station, TX).

Descriptive statistics (proportions, means, standard deviations [*SD*s]) were used to explore baseline demographic, medical and psychosocial characteristics. Chi-square analyses and independent samples t-tests examined differences between the BEHOLD-8 and BEHOLD-16 samples. Given that there were no significant differences in demographic or medical variables between the two groups (Table 1), we combined the two samples in all subsequent analyses. Additionally, we visualized MVPA individual trajectories using individual line graphs to explore the distributions of treatment responses before beginning LGCM analyses. The individual line graphs revealed that time was not linear and prompted us to include the quadratic term, ‘time^2^’ (which was significant in the regression model), in addition to ‘time,’ in our regression model.

**Table 1.** Baseline participant characteristics and measure scores.^a^^,^^b^

Characteristic/measure	Overall (*n* = 47)	BEHOLD-8 (*n* = 26)	BEHOLD-16 (*n* = 21)	Test statistic	*P*-value
*Sociodemographic characteristics*					
Age (mean [*SD*])	66.1 (10.1) M: 65.9 (12.1) F: 66.4 (8.0)	65.8 (10.9)	66.6 (9.3)	*t* = −0.28	.78
Male sex	23 (48.9)	12 (46.2)	11 (52.4)	*X*^2 ^= 0.18	.67
Non-Hispanic White	38 (80.9)	22 (84.6)	16 (76.2)	*X*^2 ^= 0.53	.47
Married	31 (65.9)	9 (34.6)	7 (33.3)	*X*^2 ^= 0.01	.93
Employed full-time	24 (51.1)	14 (53.9)	10 (47.6)	*X*^2 ^= 0.18	.67
>Four-year college education	32 (68.1)	18 (69.2)	14 (66.7)	*X*^2 ^= 0.04	.85
*Medical characteristics and measures*					
Neuropathy	10 (21.3)	5 (19.2)	5 (23.8)	*X*^2 ^= 0.15	.70
Nephropathy	13 (27.7)	9 (34.6)	4 (19.1)	*X*^2 ^= 1.41	.24
Hyperlipidemia	45 (95.7)	24 (92.3)	21 (100.0)	*X*^2 ^= 1.69	.19
Hypertension	43 (91.5)	24 (92.3)	19 (90.5)	*X*^2 ^= 0.05	.82
Coronary artery disease	14 (29.8)	7 (26.9)	7 (33.3)	*X*^2 ^= 0.23	.63
BMI at baseline (M [*SD*])	30.7 (5.0)	31.2 (5.9)	30.1 (3.8)	*t* = 0.75	.46
A1C at baseline (M [*SD*])	7.4 (1.2)	7.3 (1.2)	7.5 (1.2)	*t* = −0.48	.63
Charlson comorbidity index (M [*SD*])	4.2 (1.6)	4.2 (1.8)	4.3 (1.5)	*t* = −0.28	.78
Physical Function PROMIS-PF (M [*SD*])	92.0 (7.2)	92.0 (8.7)	92.1 (4.8)	*t* = −0.06	.95
Pain disability PDI (M [*SD*])	8.0 (9.1)	8.5 (10.6)	7.3 (7.7)	*t* = 0.45	.65
*Medications at enrollment*					
Non-insulin diabetes medications (e.g. metformin, sulfonylureas)	44 (93.62)	23 (88.5)	21 (100.0)	*X*^2 ^= 2.59	.11
Insulin	4 (8.5)	2 (7.7)	2 (9.5)	*X*^2 ^= 0.05	.82
Antidepressant	10 (21.3)	7 (26.9)	3 (14.3)	*X*^2 ^= 1.11	.29
Anxiolytic	4 (8.5)	2 (7.7)	2 (9.5)	*X*^2 ^= 0.05	.82
*Psychosocial measures*					
Optimism LOTR (M [*SD*])	19.6 (6.7)	23.0 (4.8)	15.4 (6.3)	*t* = 4.63	.00**
Positive affect PANAS (M [*SD*])	34.9 (6.0)	34.6 (6.0)	35.3 (6.2)	*t* = −0.40	.69
Anxiety HADS-A (M [*SD*])	5.9 (4.1)	5.5 (3.9)	6.3 (4.4)	*t* = −0.68	.50
Depression HADS-D (M [*SD*])	3.7 (3.5)	3.8 (3.0)	3.6 (4.2)	*t* = 0.18	.86

^a^ *N* (%) unless noted. ^b^Test statistics are chi-square or F statistic from one-way ANOVA.

**p < .01.

### LGCM analyses of MVPA

LGCM allows for the identification of subgroups within a larger population who share similar longitudinal trajectories and characteristics. Each latent growth curve has an identified slope and *y*-intercept. In these analyses, the *y*-intercept represented average minutes of daily MVPA at Time 1. The slope indicated the change in MVPA over time as measured at the three time points: baseline/before each intervention (Time 1, coded as 0 for analysis), immediately following the interventions (Time 2, coded as 1 for analysis; eight weeks for BEHOLD-8 and 16 weeks for BEHOLD-16), and eight weeks post-interventions (Time 3, coded as 2 for analysis).

To determine how many latent profiles were appropriate for our sample, we used the Stata generalized linear latent and mixed models (GLLAMM) code (Duncan & Duncan, 2009; Rabe-Hesketh, Skrondal, & Pickles, 2004) to estimate fit statistics for 2, 3, and 4 profile solutions using maximum likelihood estimation. GLLAMM is a Stata command to fit multilevel and latent variable models, including longitudinal/repeated measures data. It is able to include specification of the growth and change functions over time, using maximum likelihood estimation (Duncan & Duncan, 2009; Rabe-Hesketh et al., 2004). In the present analysis, we used GLLAMM following the specifications in the manual (found at gllamm.org), including ‘id’ as the grouping variable, the number of random effects set to 2 (allowing the intercept and slope to vary). Additional details about the Stata code can be obtained from the authors.

Because this was an exploratory data analysis, we used a combination of statistical and scientific criteria to compare fit statistics. If the lowest Akaike Information Criterion (AIC) and Bayesian Information Criterion (BIC) values were incongruent, we planned to compare both the AIC and BIC, as well as take into consideration the sample size in each profile to estimate the most scientifically relevant number of profiles.

We then ran the GLLAMM code first with time, and then including the quadratic term, time^2^. The first model, which did not use the quadratic (time^2^) term, identified the unique *y*-intercepts and slopes of the three latent growth curves, and it also identified the probability of an individual being assigned to each profile. The second model included time and time^2^. Rather than including additional covariates in the model with this relatively small sample, the participant characteristics (sociodemographic, medical, and psychosocial) of the identified latent profiles were compared using 1-way ANOVAs for continuous variables and chi-squared for categorical variables. Given the small size of profile 3, we also ran these between-group comparisons using the corresponding non-parametric Kruskal–Wallis tests for continuous variables and found that the *p*-values were similar. Therefore, we present the parametric test statistics herein, noting where there were differences between the two types of tests’ conclusions. Characteristics included in the profile comparisons were: study (BEHOLD-8 or BEHOLD-16), age, sex, race/ethnicity, marital status, annual income, employment status, college-educated, neuropathy, retinopathy, nephropathy, hyperlipidemia, hypertension, coronary artery disease, heart failure, prescribed insulin, prescribed antidepressant, prescribed anxiolytic, prescribed non-insulin diabetes medication, Charlson age-adjusted score, BMI at baseline, hemoglobin A1C at baseline, PROMIS-PF, PDI, LOTR, HADS-A, HADS-D, PANAS. These variables were included due to their relevance to physical activity and intervention parameters.

### Ethics, consent, and permissions

Both trials received Institutional Review Board (IRB) approval from our institution prior to study initiation, they were registered on ClinicalTrials.gov (Registration#: NCT03001999 (BEHOLD-16) and NCT03150199 (BEHOLD-8)), and all participants provided written informed consent.

## Results

Of the 65 participants enrolled in the BEHOLD intervention arms, 18 participants (27.7%) were missing physical activity data at one of the three time points, and therefore 47 participants’ data were included in these analyses. Baseline sociodemographic, medical, and psychosocial characteristics are shown in Table 1. The mean age of participants was 66.1 (SD 10.1), 48.9% were men, and 80.9% were non-Hispanic White. There were no significant (*p *< .05) differences between the BEHOLD-8 and BEHOLD-16 samples in terms of sociodemographic or medical characteristics. The only significant difference (*p *< .01) between the two samples was in baseline optimism (LOT-R), on which BEHOLD-8 participants had a higher score (mean = 23.0, *SD* = 4.8) compared to BEHOLD-16 participants (mean = 15.4, *SD* = 6.3; *p *< .01). None of the other psychosocial measures were significantly different between the two samples.

*LGCM analyses of MVPA*. Test fit statistics for the 2, 3, and 4 profile solutions are reported in Table 2. The AIC and BIC for the 2, 3, and 4 profile solutions were 1167.72 and 1188.36, 1144.34 and 1173.82, and 1138.31 and 1176.64, respectively (Table 2). Based on the lowest BIC and the fact that the 3 profile model was scientifically more interpretable in terms of sample sizes, we’ve chosen to use and present the 3 profile model.

**Table 2.** Fit statistics for LGCM profiles.

Number of solutions	AIC	BIC
2	1167.72	1188.36
3	1144.34	1173.82
4	1138.31	1176.64

Note: AIC: Akaike information criteria; BIC: Bayesian information criteria

The first GLLAMM showed that both time and time^2^ were significantly associated with the change in MVPA over time. In the sample as a whole, the *y*-intercept (i.e. the average MVPA minutes at Time 1) was 10.5 (CI: 6.7–14.3) minutes of MVPA per day. The linear slope (i.e. change over the three time points) was a positive increase of 18.0 (CI: 10.1–25.8) minutes per time point, while the quadratic slope was −7.2 (CI: −10.7 to −3.6) minutes per time point, indicating an increase at Time 2 followed by a decrease at Time 3.

**Figure 2.**
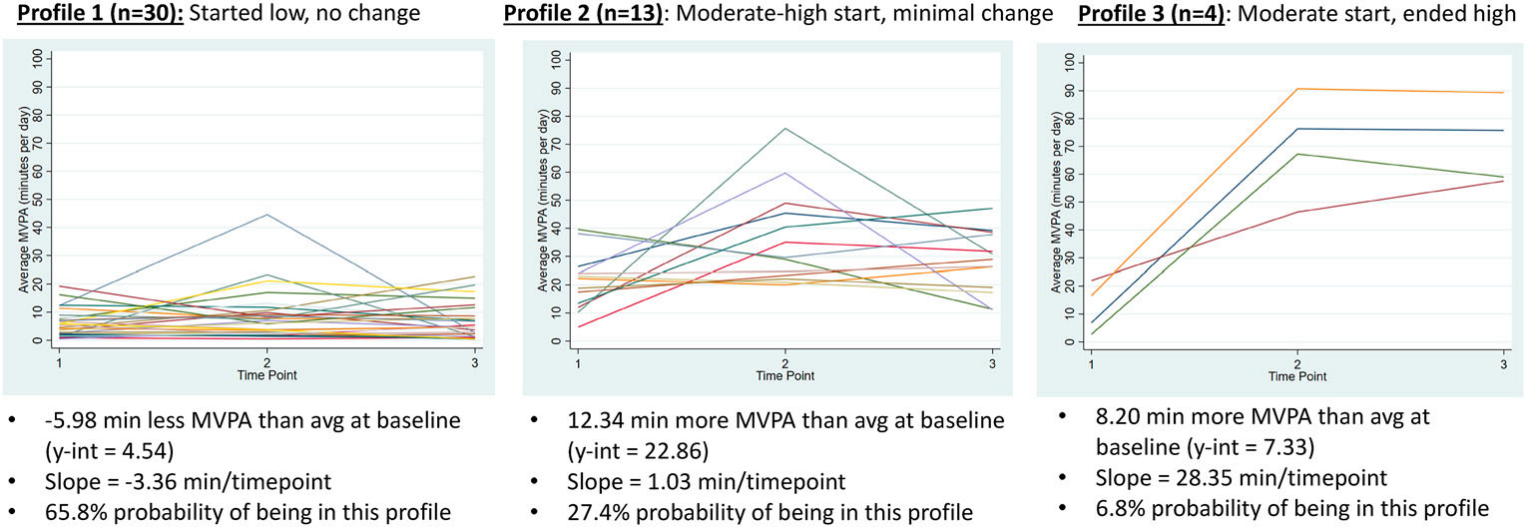
Latent profiles of MVPA: Individual growth curves grouped by profile.

The first GLLAMM also showed the unique y-intercepts and slopes for the three latent profiles, to which the researchers added descriptive names (Table 3 and Figure 2). Profile 1 (‘started low, no change’) had a *y*-intercept of 4.5 min of MVPA per day and a linear slope of −3.4 min per time point, indicating that this subgroup started 6 min lower than average and decreased over time on average. There was a 65.8% chance of a participant being in this subgroup (n = 30). Profile 2 (‘moderate-high start, minimal change’) had a *y*-intercept of 22.9 min of MVPA per day and a linear slope of 1.0 min per time point, meaning that members of this group started 12.3 min above the overall average and had a minor increase over time. There was a 27.4% chance of being in this subgroup (*n* = 13). Finally, profile 3 (‘moderate start, ended high’) had a *y*-intercept of 20.7 min of MVPA per day with a linear slope of 28.3 min per time point. Although profile 3 started with a *y*-intercept slightly lower than that of profile 2 (and 8.2 more minutes of MVPA than the average baseline), they displayed the largest increases in MVPA over the course of the study. There was a 6.8% chance to being assigned to this profile (*n* = 4).

**Table 3.** LGCM physical activity profile results.

Profile	*y*-Intercept (profile mean MVPA minutes)	Slope (change in minutes of MVPA/time point)	Time 2 profile mean MVPA minutes	Time 3 profile mean MVPA minutes	Chance of profile membership
1 ‘started low, no change’ (*n* = 30)	4.5	−3.4	1.1	−2.3	65.8%
2 ‘moderate-high start, minimal change’ (*n* = 13)	22.8	1.0	23.8	24.8	27.4%
3 ‘moderate start, ended high’ (*n* = 4)	20.7	28.3	49	77.3	6.8%

The participant sociodemographic, medical, and psychological characteristics across the three profiles are presented in Table 4. Parametric test results are presented for ease of interpretation, with indications where conclusions changed using non-parametric tests. Confidence intervals are presented for variables with significant between-group differences. Overall, being male, younger, and having fewer medical and psychiatric comorbidities led to being in a more active profile (e.g. 2 or 3). There was a significant main effect of age on MVPA profile membership (*F*(46) = 5.6, *p *= .0067), whereby people in profiles 2 and 3 (doing more physical activity over time) were younger than those in profile 1 (more sedentary). Males were more likely to be in profiles 2 or 3 than females, and more females were in profile 1 (*X*^2 ^= 8.1, *p = *.018). There was also a significant main effect of the age-adjusted Charlson comorbidity index, a measure of medical comorbidities, on MVPA profile membership (*F*(46) = 5.14, *p = *.0099), whereby people in profiles 2 and 3 had less of a comorbidity burden than those in profile 1. Depression and anxiety both had significant main effects on MVPA profile membership. Taking an antidepressant was significant (*X*^2 ^= 7.2, *p = *.027), with profile 1 containing all of the people taking antidepressants (none in profiles 2 or 3). Physical functioning was significantly worse in profile 1 than profiles 2 and 3 on the non-parametric test (*X*^2 ^= 7.1, *p = *.028), but only marginally significant on the parametric test (*F*(46) = 2.6, *p *= .087). Finally, HADS-Anxiety scores had a significant main effect on MVPA profile membership on the parametric test (F(46) = 3.43, *p = *.041), with people in profiles 2 or 3 scoring lower than those in profile 1, but this finding became marginally significant using the non-parametric test (*X*^2 ^= 5.7, *p = *.057).

**Table 4.** Sociodemographic, medical, and psychosocial characteristics of the three MVPA profiles.

Characteristic/measure	Overall (*n* = 47)	Profile 1 (*n* = 30)	Profile 2 (*n* = 13)	Profile 3 (n = 4)	Test statistic	*P*-value
*Sociodemographic characteristics N* (*SD*) or frequency (% of profile membership)						
Age (mean [*SD*])	66.1 (10.1)	69.5 (8.2) CI: 66.1–72.9	59.4 (9.2) CI: 54.2–64.5	63.2 (16.1) CI: 53.9–72.5	*F* = 5.6	<.01**
Male sex	23 (48.9%)	10 (33.3%) CI: 17.3–52.8	10 (76.9%) CI: 46.2–95.0	3 (75%) CI: 19.4–99.4	*X*^2 ^= 8.1	.02*
Non-Hispanic White	38 (80.9%)	29 (96.7%)	13 (100%)	4 (100%)	*X*^2 ^= 0.58	.75
Married	31 (65.9%)	19 (63.3%)	10 (76.9%)	2 (50%)	*X*^2 ^= 7.0	.32
Employed full-time	24 (51.1%)	10 (43.5%)	11 (47.8%)	2 (50%)	*X*^2 ^= 10.5	.10
>Four-year college education	32 (68.1%)	20 (66.7%)	7 (53.8%)	4 (100%)	*X*^2 ^= 12.3	.26
*Medical characteristics and measures*						
Neuropathy	10 (21.3%)	7 (23.3%)	1 (7.7%)	2 (50%)	*X*^2 ^= 3.5	.18
Nephropathy	13 (27.7%)	10 (33.3%)	2 (15.4%)	1 (25%)	*X*^2 ^= 1.5	.48
Hyperlipidemia	45 (95.7%)	29 (96.7%)	13 (100%)	3 (75%)	*X*^2 ^= 4.9	.09
Hypertension	43 (91.5%)	28 (93.3%)	12 (92.3%)	3 (75%)	*X*^2 ^= 1.5	.46
Coronary artery disease	14 (29.8%)	10 (33.3%)	3 (23.1%)	1 (25%)	*X*^25.1 ^= .50	.78
BMI at baseline (M [*SD*])	30.7 (5.0)	31.2 (5.6)	30.7 (3.3)	27.3 (4.0)	*F* = 1.1	.34
A1C at baseline (M [*SD*])	7.4 (1.2)	7.4 (1.1)	7.6 (1.5)	6.7 (.86)	*F* = .89	.42
Charlson comorbidity index (M [*SD*])	4.2 (1.6)	4.7 (1.4) CI: 4.2–5.3	3.2 (1.3) CI: 2.4–4.1	3.5 (2.6) CI: 2.0–5.0	*F* = 5.1	.01*
Physical physical function PROMIS-PF (M [*SD*])	92.0 (7.2)	90.4 (7.1) CI: 87.8–92.9	95.6 (4.1) CI: 91.7–99.5	92.5 (12.4) CI: 85.5–99.5	*F* = 2.6	.09^(*)^
Pain disability PDI (M [*SD*])	8.0 (9.1)	9.1 (9.8)	6.4 (8.1)	4.2 (4.3)	*F* = .77′	.47
*Medications at enrollment*						
Non-insulin diabetes medication (metformin)	41 (87.2%)	25 (83.3%)	13 (100%)	3 (75%)	*X*^2 ^= 2.8	.24
Insulin	4 (8.5%)	3 (10.0%)	1 (7.7%)	0 (0%)	*X*^2 ^= .47	.79
Antidepressant	10 (21.3%)	10 (33.3%) CI: 17.3–52.8	0 (0%) CI: n/a	0 (0%) CI: n/a	*X*^2 ^= 7.2	.03*
Anxiolytic	4 (8.5%)	2 (6.7%)	1 (7.7%)	1 (25%)	*X*^2 ^= 1.5	.46
*Psychosocial measures*						
Optimism LOTR (M [*SD*])	19.6 (6.7)	18.9 (7.0)	19.6 (6.1)	24.5 (4.7)	*F* = 1.2	.30
Positive affect PANAS (M [*SD*])	34.9 (6.0)	34.6 (5.3)	36.3 (8.1)	32.7 (3.8)	*F* = .63	.54
Anxiety HADS-A (M [*SD*])	5.9 (4.1)	7.0 (4.4) CI: 5.5–8.4	3.8 (2.9) CI: 1.6–6.0	4.2 (2.2) CI: .28–8.2	*F* = 3.4	.04*^a^
Depression HADS-D (M [*SD*])	3.7 (3.5)	4.3 (4.0)	2.6 (2.2)	2.7 (2.4)	*F* = 1.2	.30

*p < .05, **p < .01, ^(*)^*p < *.05 on non-parametric test, ^a^*p *= .06 on non-parametric test.

CI: Confidence interval.

## Discussion

These secondary analyses identified three unique subpopulations within the larger BEHOLD intervention groups that displayed similar MVPA trajectories over the course of the 8- and 16-week intervention and subsequent follow-ups. In the sample as a whole, the linear slope for MVPA over time was positive with increases at each of the three time points, while the quadratic slope for time was negative, indicating an increase in MVPA during the intervention, followed by an overall decrease at follow-up. The LGCM analyses revealed that 36% percent of the participants showed an increase in MVPA over time while the other 57% displayed no significant change. These findings are in line with previous studies of behavioral physical activity interventions for people with type 2 diabetes, which indicate that there may be initial overall intervention effectiveness that is often followed by declines at later time points (Balducci et al., 2019; Cradock et al., 2017; Deakin, McShane, Cade, & Williams, 2005; Kirk, Mutrie, MacIntyre, & Fisher, 2003; Plotnikoff, Costigan, Karunamuni, & Lubans, 2013).

Within primary single-group outcome studies, little is known about differential responses to physical activity interventions by subgroup or population, particularly in T2D. The limited prior research has shown that there are health, demographic, psychosocial, and environmental factors that lead to different responses to physical activity interventions, for example, social support, self-efficacy, and self-regulation (King et al., 2006; Rovniak, Anderson, Winett, & Stephens, 2002). The type and intensity of physical activity interventions also appear to be an important predictor of success, such as in-person, group sessions, and those tailored for women and people of specific racial/ethnic groups (Bock et al., 2014). In community samples, correlates of physical activity such as social support, prior injuries, education level, health status are also associated with different subgroups of physical activity, in the absence of intervention (Plotnikoff, Mayhew, Birkett, Loucaides, & Fodor, 2004). These findings are in line with the present study’s findings that participants who were male, younger, and had fewer psychiatric and medical comorbidities showed greater increases in MVPA over the course of the study. While no other variables were significantly associated with MVPA profile membership, there were trends in which profile 1 tended to have worse scores than profile 3 (e.g. better functioning was related to growth in physical activity) with respect to BMI, pain disability, and optimism, which can all impact physical activity trajectory outcomes and response to interventions (Bartley, Palit, Fillingim, & Robinson, 2019; Fortier & Morgan, 2021). However, none of these findings have been explored in samples of people with T2D, which should be a key target of future tailoring of physical activity interventions.

This study had several limitations. This was a secondary analysis and was not hypothesis-driven, but rather sought to explore trajectories and subgroups in a previously conducted set of studies. It included a relatively small sample for LGCM (Kelley & Rausch, 2011) – a large sample size is typically recommended (e.g. >100) for latent modeling (Shi, DiStefano, Zheng, Liu, & Jiang, 2021) – which can introduce uncertainty into the models and conclusions. Thus, these findings must be considered exploratory and should be validated in larger studies. However, we believe this sample size, which is large for the field of positive psychology and motivational interviewing interventions at this time, is useful for designing future studies in that it can allow detection of a signal of different response profiles. There was a small number of people (4) in profile 3 that showed the greatest improvements, so most people did not increase their physical activity in a meaningful way after the intervention. It would have been ideal if more people improved so that we could more robustly assess characteristics of change and the comparisons with the other (larger) profiles, given that there were often wide confidence intervals around the point estimates in group 3. Further, people in profile 2 started out at a moderate to high activity level, so it is possible that this also reduced their degree of increase over time. The lack of long-term maintenance of physical activity, or positive results being driven by a small number of people making large changes, are common patterns for psychosocial and behavioral interventions that are persistent issues within the field (Hobbs et al., 2013). Future studies of this nature should include larger samples. We combined two datasets (BEHOLD-8 and BEHOLD-16) and made assumptions that combining the timing of assessments (e.g. time 2 post-intervention and time 3 at eight-week post-intervention follow-ups) would be equivalent. Finally, we only included participants with complete accelerometer data at all three time points, which may have led to an overestimation of physical activity change if those who stayed in the study performed better than those who dropped out.

Future versions of this PP-MI intervention might seek to target the individuals that showed the greatest benefit (e.g. younger adults, males, low anxiety, low comorbidities), and to add additional or tailored supports to people in groups that did not perform as well (e.g. women, older adults, higher comorbidities, higher anxiety). The people in profile 1 – who were less likely to change their activity – may benefit more from a longer intervention (e.g. 16 weeks), even though the eight-week intervention was more efficacious overall (Huffman et al., 2020; Huffman et al., 2021). The intervention manuals were fairly text-heavy, so it may have taken a certain level of energy or focus to read the material and engage with it fully, so perhaps people with more comorbidities had fewer resources to do so. Overall, however, physical activity as an outcome may be more amenable to change for the people in profiles 2 and 3: more likely to be male, somewhat younger, and healthier. Even in the context of a fairly intensive intervention, the people in the majority group (profile 1), who were more likely to be older, female, and with more comorbidities, seem to have more barriers to increasing activity. Larger sample sizes in future studies can help to understand this intervention’s effects compared to other studies of physical activity intervention change profiles. Potential next-step interventions could include emphases on different types of physical activity that could appeal to a more diverse or older audience, such as culturally tailored dance or movement programs and gentler exercises (e.g. yoga, tai chi). Also, successful T2D physical activity interventions tend to include multiple elements from the behavior change taxonomy, such as behavioral rehearsal, demonstrations of different activities, and instructions on how to do different activities (Cradock et al., 2017). While the present studies did emphasize action planning, another key behavior change element (Cradock et al., 2017), future tailored studies may need additional supports for the subgroups at higher risk of limited change. Overall, these findings also show a need for sustained behavior change following interventions, which may take the form of tailored text messages or other virtual supports following active intervention. Results from this study will inform the development of future targeted PA interventions for T2D.

## Supplementary Material

Supplemental Material

## Data Availability

The datasets supporting the conclusions of this article are available by contacting the corresponding author.
