# Supplementary material for: Latent growth curve modeling of physical activity trajectories in a positive-psychology and motivational interviewing intervention for people with type 2 diabetes
Source: Health Psychol Behav Med. 2022 Aug 4;10(1):713–30. doi: 10.1080/21642850.2022.2104724 (PMC9359186; doi:10.1080/21642850.2022.2104724)
Supplement: Supplemental Material [file RHPB_A_2104724_SM4630.docx]

Supplemental Table 1. Baseline comparisons between participants with complete (included) vs. incomplete (excluded from analyses) physical activity data.

| Characteristic/Measure  (mean (SD) or n (%) | Included in analyses (n=47) | Excluded from analyses (n=18) | Test Statistic | P-value |
| --- | --- | --- | --- | --- |
| *Baseline Characteristics* | | | | |
| Age (mean [SD]) | 66.1 (10.1) | 61.7 (10.3) | t = 1.6 | 0.12 |
| Male sex | 23 (48.9%) | 10 (55.0%) | X^2^= 0.23 | 0.63 |
| Non-Hispanic White | 38 (80.9%) | 10 (55.0%) | X^2^= 4.3 | 0.04* |
| Married | 31 (65.9%) | 8 (44%) | X^2^= 2.5 | 0.11 |
| Employed full-time | 24 (51.1%) | 10 (55.0%) | X^2^= 0.23 | 0.63 |
| >4-year college education | 32 (68.1%) | 6 (33.3%) | X^2^= 9.2 | 0.10 |
| Mean minutes MVPA/day | 10.5 (9.6) | 16.6 (14.7) | t=1.9 | 0.06 |

**p*<.05
